# Parental absence predicts suicide ideation through emotional disorders

**DOI:** 10.1371/journal.pone.0188823

**Published:** 2017-12-07

**Authors:** Mingchen Fu, Yan Xue, Weihe Zhou, Ti-Fei Yuan

**Affiliations:** 1 School of Education Science, Nanjing Normal University, Nanjing, Jiangsu, China; 2 Faculty of Education, the University of Hong Kong, Hong Kong, China; 3 School of Psychology, Nanjing Normal University, Nanjing, Jiangsu, China; 4 The Eye Hospital of Wenzhou Medical University, Wenzhou, China; 5 Shanghai Key Laboratory of Psychotic Disorders, Shanghai Mental Health Center, Shanghai Jiao Tong University School of Medicine, Shanghai, China; 6 Co-innovation Center of Neuroregeneration, Nantong University, Nantong, Jiangsu, China; Old Dominion University, UNITED STATES

## Abstract

The objectives of the study were to investigate the association between the parental absence and suicide ideation and to examine the roles of emotional disorders in the aforementioned relationship. Four thousand five hundred and thirteen children from rural areas in Jiangsu Province, China, participated in the study. Among the participants, 2416 were non-left-behind children (children living with both parents) and 1997 were left-behind children (children living with the absence of at least one parent). All participants responded to the Center for Epidemiological Studies Depression Scale for Children, the Multidimensional Anxiety Scale for Children, and a question regarding respondents’ suicide ideation. Results indicated that as compared with non-left-behind children, the left-behind children with both-parents absence were statistically more likely to show suicide ideation. Furthermore, all the three types parental absence—father absence, mother absence, and both-parents absence were significantly associated with negative emotional outcomes. Moreover, depression, social anxiety, and physical anxiety were shown to be significant mediators in the relationship of parental absence and suicide ideation of children. The stress of parental absence and its negative impact on children’s mental health are discussed.

## Introduction

For a long time in psychology, being separated from parents has been considered as a significant distress for children. Literature indicated that children would show negative emotions like anxiety, angry, and fear as responses of being separated from parents [1]. Along with the development of the modern society, long-term parental absence has become a considerable issue that attracts the attention of researchers [2–4]. On the one hand, increased divorce rates have been frequently reported [5]. On the other hand, large-scale labor migration has being occurring in many areas of the world, leading to a huge number of children being left in home with the absence of parental cares [3–4].

Because of the rapid urbanization of China in the recent 30 to 40 years, a considerable number of children in rural areas in China have been experiencing long-term parental absence, given their parents have migrated to urban area as rural laborers for city building [6]. These children are usually called left-behind children, a term referring specifically to the children who live at their original residence with one or both parent(s) having left home and migrated to other places for more than 6 months [7]. In the recent decade, much empirical research paid particular attention to investigate the psychological status of the left-behind children in China, and such research contributed greatly to the literature on the relationship between parental absence and child development. Based on previous research evidence, as compared with non-left-behind children, left-behind children have shown to be more likely to have emotional disorders such as depression and anxiety [7–8] and behavior dysfunctions such as bully behaviors and internet addiction [9–10] and to perform poorer in cognitive tests [9–11].

Even though a good number of factors regarding the mental health of left-behind children have been explored by previous empirical research, there have been no study investigated the suicide ideation among left-behind children. Suicide ideation, as suggested by literature, is the thoughts of “killing or harming oneself” [12](p1). Previous research has evidenced that suicide ideation is significantly associated with emotional disorders, such as depression and anxiety [13–14]. Considering that children with parental absence have been widely reported to be under high risk of getting emotional disorders (see previous Discussion), it was reasonable to believe that a considerable proportion of left-behind children would be suffering from suicide ideation.

According to some surveys conducted in recent years, suicide has become one of the top causes for death of children and adolescents throughout various areas of the world [15–16]. With regard to the situation in China, Tan, Xia, and Reece randomly surveyed 13,822 children from 13 different cities across China and found the prevalence of suicide ideation among these children were approximately 30.09% [12]. Therefore, the high rate of suicide and suicide ideation has been a serious issue that threatened the health of children in China. Considering the large number of left-behind children and the potential high risk of possessing suicide ideation of children under the stress of parental absence, deep investigations on the suicide ideation of left-behind children was needed.

Based on a sample consisting of both left-behind children and non-left behind children (children living with both parents) in rural area of China, the study was intended to investigate the association between the parental absence and suicide ideation and the role of emotional disorders in such relationship. Three hypotheses were made as follows:

1. As compared with non-left-behind children, left-behind children would be at statistically higher risk of having suicide ideation;
2. Left-behind children would suffer from statistically higher level of negative emotions as compared with their non-left-behind peers;
3. Emotional disorders would significantly strengthen the associations of children’s status of parental absence with their suicide ideation.

## Method

### Participants

Participants of the current study were 4513 children from rural areas in Yancheng, China. Before we conducted our study, we sent our study proposal to all primary and middle schools in rural areas in Yancheng, including 55 primary schools and 67 middle schools. Yancheng, is an area that is relatively backward in economy in Jiangsu Province, China and many adults in rural areas in Yancheng had migrated to cities to seek livelihoods, with their children left at home. Nineteen schools, including 12 primary schools and 7 middle schools responded and agreed to help coordinate recruiting participants in their schools for our study. Under the schoolteachers’ help, our researchers went to all classes from Grade 3 to Grade 9 in each school, expressing the study purpose to the students and letting the students to bring the consent form to their guardians. We excluded students under Grade 3 because these students did not have the basic reading ability to answer our questionnaire. We had also excluded the children who had mental deficiencies based on the information provided by the schoolteachers. The participation of our study was entirely voluntary. We involved the students as our participants only after we got the signature of their guardians and their own. Response rates in the 19 schools ranged from 82.7% to 96.3%. Ethical approval for the study was obtained from the Ethics Committee of Nanjing Normal University (China).

Finally, 4513 children participated in our study. Ages of the participants were from 9 to 17 years old (*M* = 12.38). Among these children, 8.3% were at Grade 3, 17.7% were at Grade 4, 18.1% were at Grade 5, 17.3% were at Grade 6, 16.0% were at Grade7, 13.5% were at Grade 8, and 8.6% were at Grade 9 students. Moreover, 52.7% children were females and 47.3% were males. Among the children that participated in the current study, there were 2416 (53.5%) non-left-behind children and 1997 (46.5%) left-behind children. Furthermore, among the left-behind children, 1003 children were living only with mother (father absence group), 132 were living only with father (mother absence group), 962 were living without the care of either parent (both-parents absence group).

### Measures

All participants responded to a questionnaire comprised of a) a range of questions concerning demographic information, including age, gender, school, grade, social economic status (SES), and the status of parental presence, b) the Center for Epidemiological Studies Depression Scale for Children (CES-DC) [17], c) the Multidimensional Anxiety Scale for Children (MASC) [18], d) a question regarding respondents’ suicide ideation. The SES of children was assessed by asking the children that which financial level of their family was at in comparison with fellow students—lower than average, average, or higher than average [19]. To detect the suicide ideation, children were asked by one question that whether or not they had the thoughts of killing themselves in the past two weeks [20–21]. All the inventories used in our study were translated into Chinese before being administered to participants. We performed the translation and back-translation process to ensure that the contents of the inventories were accurately translated (see S1 File for our Chinese version questionnaire).

The study utilized the CES-DC to evaluate children’s depression level [17]. The CES-DC has 20 items and all times are scored by a 4-point Likert scale, with 1 indicating “not at all” and 4 indicating “a lot”. Examples of the items are: “during the past one week, I felt like I was too tired to do things” and “during the past one week, I had a good time (reversely scored)”. The inventory was a uni-dimentional scale and the higher scores represents increasing level of depression. The CES-DS has been broadly adopted in previous empirical studies and been reported with satisfactory psychometric properties [22–24]. In the current study, the reliability of the inventory was .87.

The MASC was adopted to test children’s status of anxiety [18]. The MASC consists of 39 items, which are divided into four subscales—social anxiety, harm avoidance, isolation anxiety, and physical anxiety. Examples of items are: “I worry about other people laughing at me” (social anxiety), “I get scared when my parents go away” (isolation anxiety), “I try to do everything exactly right” (harm avoidance), and “I get dizzy or faint feelings” (physical anxiety). The MASC has been widely adopted to test the anxiety status of children in empirical studies and been validated across cultures [18, 25–26]. In the current study, the four-dimension structure of the MASC was further validated by exploratory factor analysis. It was found that items corresponding to the six types of anxieties were successfully identified from the MASC and the four factors accounted for 47.95% variance in the data. Furthermore, the reliabilities of the inventory were .83, .65, .71, and .87, respectively, for the subscales of social anxiety, harm avoidance, isolation anxiety, and physical anxiety.

### Data analyses

With respect to data analyses, first, psychometric properties the CES-DC and the MASC were evaluated by exploratory factor analysis and Cronbach’s alpha test. Second, descriptive statistics were carried out to investigate the statistical distributions of the continuous variables (e.g., depression, social anxiety, harm avoidance) and to test the prevalence of suicide ideation among children with different status of parental absence. Third, logistic regression was conducted to test the relationship between parental-absence status and suicide ideations with demographic factors being controlled. Fourth, ANOVA and post hoc analyses with Bonferroni correction were performed to examine the difference of depression and anxiety levels across children with different parental-absence status. Fifth, simple mediation models were established for evaluating the roles of depression and anxiety in the association of parental-absence status to suicide ideation [27–28]. All data analyses were performed in SPSS 19. The SPSS PROCESS was utilized particularly to test the indirect effects of parental-absence status to suicide ideation through depression or anxiety [28].

## Findings

The descriptive statistics of depression and four indicators of anxiety and their inter-correlations are in Table 1. Given that the absolute values of skewness and kurtosis of all variables were < 2.0 and <7.0, respectively, all the continuous variables were considered as normally distributed [29].

**Table 1:** Descriptive statistics and correlations between key variables.

	Mean	SD	Skewness	Kurtosis	1	2	3	4
1 Depression	1.99	.53	.48	-.11				
2 Social anxiety	2.60	.94	.18	-.72	.51**			
3 Isolation anxiety	2.44	.87	.39	-.31	.28**	.44**		
4 Harm Avoidance	3.54	.89	-.30	-.38	-.07**	.11**	.19**	
5 Physical anxiety	1.84	.61	.74	.34	.63**	.47**	.25**	-.02

Note.

** *p* <.01.

**Fig 1:**
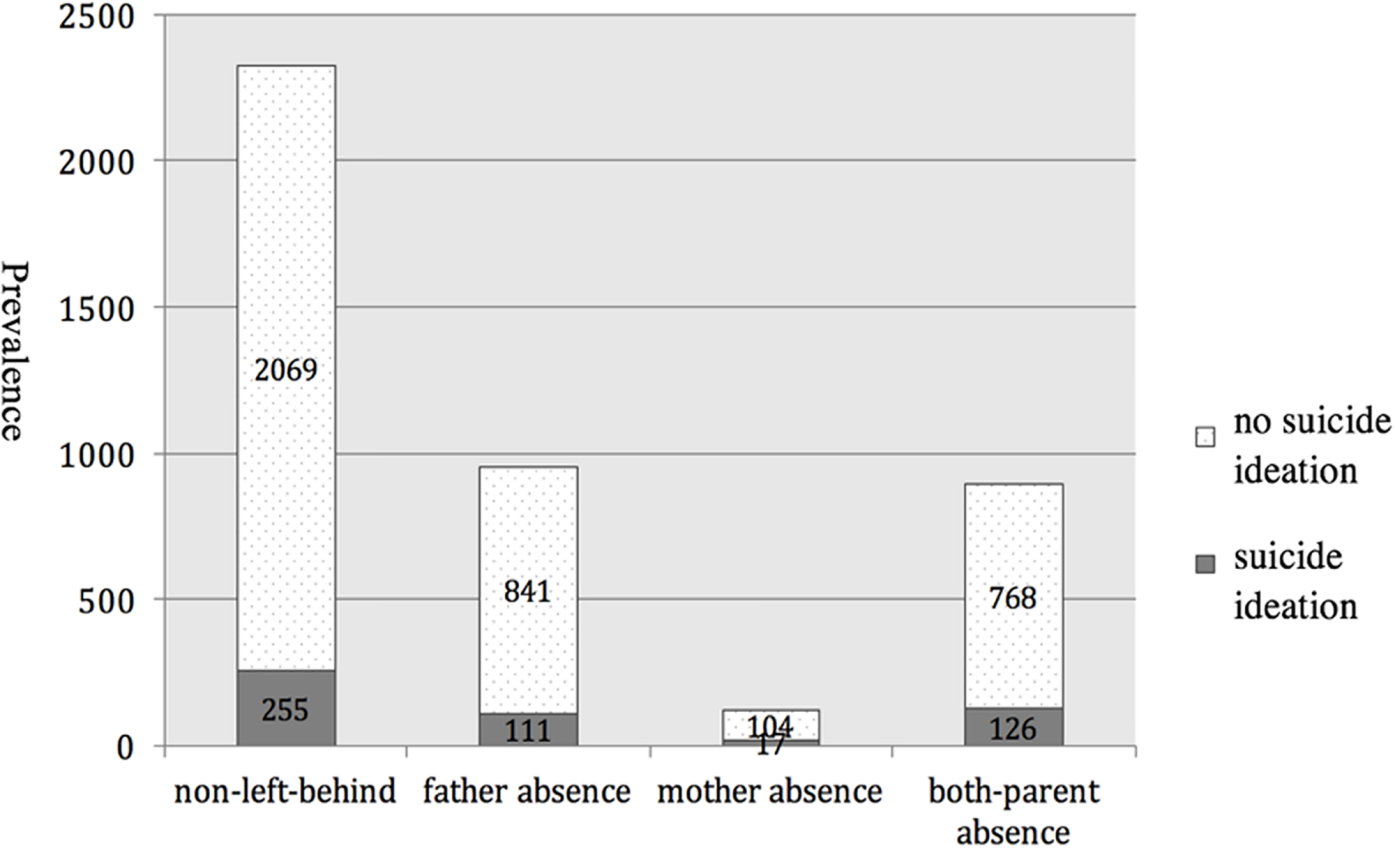
The prevalence of suicide ideation across children with various status of parental absence.

Fig 1 presents the prevalence of suicide ideation among children with different status of parental absence. As shown in the figure, 255 out of the 2324 (11.0%) non-left-behind children, 111 out of the 925 (11.7%) children with father absence, 17 out of the 121 (14.0%) children with mother absence, and 126 out of the 894 (14.1%) children with both-parents absence reported suicide ideations. Therefore, it was observed from the descriptive data that left-behind children prevalently showed higher rate of suicide ideation than non-left-behind children.

Logistic regressions results showed a clearer picture further concerning the comparisons between the prevalence of suicide ideation among left-behind children and that of non-left-behind children. As presented in Table 2, with demographic factors being controlled and non-left-behind children set as reference, the status of both-parents absence showed significant association with suicide ideation while the other two status of parental absence did not. Specifically, it was found that children with both-parents absence would be 1.35 (95% CI = (1.06, 1.71)) times more likely to have suicide ideations than non-left-behind children.

**Table 2:** Predicting suicide ideation from status of parental absence.

	OR	Sig.	95% C.I. OR	
			Lower	Upper
age	1.03	.074	1.00	1.07
*family income level*				
low (reference)		.015		
mediate	.86	.226	.67	1.10
high	1.38	.092	.95	2.02
*status of parent absence*				
non-left-behind (reference)		.113		
father absence	1.10	.469	.85	1.41
mother absence	1.22	.506	.68	2.19
**both-parents absence**	**1.35**	.**016**	**1.06**	**1.71**
Constant	.09	.000		

*Note*. Age, family income level, and gender were controlled in the model; logistic regression was performed with forward method (likelihood ratio test).

The associations of parental absence status with the depression and anxiety were tested by ANOVA. As shown in Table 3, the status of parental absence was statistically associated with depression and all four indicators of anxiety. Post hoc analyses further indicated that, first, children with father absence (*M* = 2.01) and both-parents absence (*M* = 2.06) suffered from statistically higher level of depression than non-left-behind children (*M* = 1.95); second, children with father absence (*M* = 2.67) and both-parents absence (*M* = 2.72) showed statistically higher level of social anxiety than non-left-behind children (*M* = 2.52); third, children with both-parents absence (*M* = 3.48) were scored significantly lower in the subscale of harm avoidance as compared with non-left-behind children (*M* = 3.58); fourth, children with mother absence (*M* = 1.95) and those with both-parents absence (*M* = 1.80) presented statistically higher level of physical anxiety than their non-left-behind peers (*M* =); fifth, children with father absence (*M* = 2.53) suffered from higher level of isolation anxiety in comparison with non-left-behind children (*M* = 2.39).

**Table 3:** The associations of status of parental absence with emotional outcomes.

dependent variable	Type III Sum of Squares	df	Mean Square	F	Sig.	Partial Eta Squared
depression	9.52	3	3.17	11.49	.000	.01
social anxiety	33.18	3	11.06	12.73	.000	.01
harm avoidance	8.48	3	2.83	3.57	.014	.00
physical anxiety	10.44	3	3.48	9.32	.000	.01
isolation anxiety	17.51	3	5.84	7.82	.000	.01

Considering that in comparison with non-left-behind children, the children with both-parents absence showed significant relative direct effects when being associated to suicide ideation and negative emotions, respectively, mediation models were established further to test the roles of emotional disorders in the relationship between parental-absence status and suicide ideation (see Table 4). As suggested by Hayes and Preacher, to assess mediation model with multicategorical predictor, all the categories can be transfer to independent dummy variables and be controlled in the model as covariates [30]. Therefore, for each mediation model, we examined the mediating effect of one emotional factor (e.g., depression, social anxiety) in the relationship of both-parents absence to suicide ideation with non-left-behind children being set as reference and the other status of parental absence being statistically controlled. As presented in Table 4, depression (indirect effect = .155, 95% CI (= .090, .222)), social anxiety (indirect effect = .115, 95% CI (= .070, .168)), and physical anxiety (indirect effect = .117, 95% CI (= .066, .175)) were respectively shown to be significant mediator in the relationship of both-parents absence and suicide ideation. Moreover, it was noted that the significant direct effect of parental absence status to suicide ideation was completely disappeared after any of aforementioned three emotional factors being controlled in the model.

**Table 4:** Mediating effects of emotional factors in the relationships of parental-absence status to suicide ideation.

Mediator	a	b	c′	ab	95% CI of ab
depression	.104***	1.492***	.090	.155***	.090, .222
social anxiety	.189***	.605***	.136	.115***	.070, .168
harm avoidance	-.097	-.074	.203	.007	-.002, .022
physical anxiety	.109***	1.075***	.134	.117***	.066, .175
isolation anxiety	0.046	.271***	.201	.013	-.004, .035

*Note*.

a = the effect of both-parents absence on proposed mediator.

b = the effect of proposed mediator on suicide ideation when the status of both-parents absence is controlled.

c’ = the effect of both-parents absence on suicide ideation when the proposed mediator is controlled.

ab = the indirect effect of both-parents absence on suicide ideation through the proposed mediator. In each mediation model, both-parents absence was set as independent variable, suicide ideation was set as dependent variable, non-left-behind children was set as reference, the other statuses of parental absence were controlled as covariates.

*** *p* <.*001*.

## Discussion

The study was the first study that investigated the association between parental-absence status and suicide ideation among left-behind children. Consistent with Hypothesis 1, it was found that children with both-parents absence were at much higher risk of having suicide ideation as compared with non-left behind children. Based on a review on literature, it was noted that most studies on left-behind children did not distinguish the different statuses of parental absence (e.g., father absence, mother absence, both-parent absence) of these children [9–10]. Results of the current study indicated that only children with both-parents absence were significantly more likely to have suicide ideation than non-left-behind children, while the left-behind children with only-one-parent absence were not. Therefore, it can be implied that even if among left-bind children, different parental status would be associated differently with various psychological status.

One previous study investigated the suicide ideation of a group of children whose parents had separated or divorced [5]. This study showed that, the group of children who lived with only one parent did not show significantly higher rate of suicide ideation than their peers. Thus, given that the current study found the significant relative effect concerning the association between both-parents absence and suicide ideation as compared with non-left-behind children, it may imply that children with both-parents absence suffers the larger stress and are at the higher risks of getting metal disorders than those living with only one parent.

Furthermore, it was found that, consistent with Hypothesis 2, the level of depression and anxiety significantly distinct between left-behind children and non-left-behind children. Particularly, finding showed that all the three types of parental absence—father absence, mother absence, and both-parents absence were statistically associated with at least one of indicators of emotional disorder. Traditionally, researchers tended to focus exclusively on the negative impact of child-mother separation on children’s mental health and behaviors [31–33], while rarely paid attention to the negative influence of father-child separation on children. Nevertheless, evidence from the current study showed that children with long-term absence of father had significantly higher level of depression, social anxiety, and isolation anxiety as compared with their peers living with both parents. Therefore, findings of the current study implied that the absence of father could also be a salient detrimental factor for children’s mental health.

Finally, it was shown that depression, social anxiety, and physical anxiety significantly mediated the relationship of parental absence status to suicide ideation. Thus, Hypothesis 3 was also supported by findings of the current study. Moreover, it was found that the significant association of both-parents absence and suicide ideation was disappeared when any of the aforementioned three mediators were controlled. Therefore, based on findings of the current study, emotional disorders are likely to be the main reason of the relatively high risk of suicide ideation children with both-parents absence.

## Conclusion, implications, and limitations

With a large sample of children from rural areas in Yancheng, China, the current study investigated the relationship between parental absence status and suicide ideation and the role of emotional disorders in such relationship The study is the first study that detected the statistical relationship between parental absence and suicide ideation of children, and that revealed the significant role of negative emotional factors (i.e., depression, anxiety) in the association of parental absence with suicide ideation. Specifically, it was found that, as compared with non-left-behind children, the left-behind children with both-parents absence were statistically more likely to show suicide ideation. Furthermore, all the three types parental absence—father absence, mother absence, and both-parents absence were significantly associated with negative emotional outcomes. Finally, depression, social anxiety, and physical anxiety were shown to be significant mediators in the relationship of parental absence and suicide ideation of children.

In the modern society, parental absence is a worldwide pervasive issue concerning child development, no matter if it is caused by broken marriage or labor migration [3, 34]. Therefore, some practical suggestions can be drawn from the findings of our study. First, teachers and caregivers should be aware of the significant association of parental absence with negative emotional factors; thereby, teachers and caregivers could pay adequate attention to the mental health status of children who are suffering from parental absence. Particularly, previous research evidence suggested that supportive teacher-student relationship plays essential role in the development of at-risk students [35]. Thus, considering that left-behind children are at higher risk of having emotional disorders, it is suggested that schoolteachers should spend extra time understanding the specific psychological needs of the students with parental absence. For example, teachers are expected to have a regular one-to-one talk with these students, getting to know their study and living status in a timely manner. Moreover, teachers are recommended to visit the families of these students, in order to get a better understanding on the living conditions of the students and what kind of supports are needed. Third, parents who do not live with their children are also encouraged to pay attention to their children’s psychological needs and do their best to build up close relationships with their children. Even though these parents do not live together with their children physically, they could still show concerns to their children through multiple ways, such as making phone/video calls and writing letters, letting their children feel supports from them in a psychological sense.

Two limitations of the study should be noted. First, even though our study was conducted based on a large sample, we only involved the schools that agreed to participate in our study; therefore, some bias may be caused due to the sampling method. Moreover, in the study, we examined the prevalence of suicide ideation within each different parental-absence group as a preliminary exploration of our data; however, it should be noted that results of the prevalence estimation cannot be generalized to the larger population given the way of our sampling. Second, our study is a cross-sectional study, by which causality conclusions cannot be drawn. Therefore, in future research, longitudinal study designs are expected to be adopted to investigate the relationship between parental absence and suicide ideation further.

## Supporting information

S1 File Chinese-version inventories used in the study. (DOCX)

S2 File Data used in the study analyses. (XLSX)
