# Supplementary material for: Parental absence predicts suicide ideation through emotional disorders
Source: PLoS One. 2017 Dec 7;12(12):e0188823. doi: 10.1371/journal.pone.0188823 (PMC5720745; doi:10.1371/journal.pone.0188823)
Supplement: S1 File — (DOCX) [file pone.0188823.s001.docx]

Appendix 1

*Chinese-Version Inventories Used in the Study*

**CES-DC**

下面是一些对于心情或生活状态的描述。请你仔细阅读每一句话，想想你在**过去这一个星期内**是否感受到这样的状态，并在每个句子后面的1-4数字上圈选一个相应的数字。**“1”表示从没有感受到，“4”表示经常感受到。**

|  | 从不 | 偶尔 | 有时 | 经常 |
| --- | --- | --- | --- | --- |
| 1. 一些以前不会让我烦恼的事情开始让我感到烦恼。 | 1 | 2 | 3 | 4 |
| 1. 我觉得不想吃东西，没有饿的感觉。 | 1 | 2 | 3 | 4 |
| 1. 我无法开心起来，即使家人和朋友们试图帮助我让我感到好过。 | 1 | 2 | 3 | 4 |
| 1. 我觉得我和其他同学一样好。 | 1 | 2 | 3 | 4 |
| 1. 我感到无法集中注意力在我所做的事情上。 | 1 | 2 | 3 | 4 |
| 1. 我感到沮丧、不开心。 | 1 | 2 | 3 | 4 |
| 1. 我感到很累，所以无法学习或做事情。 | 1 | 2 | 3 | 4 |
| 1. 我觉得会有什么好的事情发生。 | 1 | 2 | 3 | 4 |
| 1. 我觉得我以前做的事情都没有一个好结果。 | 1 | 2 | 3 | 4 |
| 1. 我感到害怕。 | 1 | 2 | 3 | 4 |
| 1. 我的睡眠不如从前那么好。 | 1 | 2 | 3 | 4 |
| 1. 我感到开心。 | 1 | 2 | 3 | 4 |
| 1. 我比往常更安静、不愿说话。 | 1 | 2 | 3 | 4 |
| 1. 我感觉到孤独， 感觉自己没有朋友。 | 1 | 2 | 3 | 4 |
| 1. 我觉得我认识的同龄人都不太友好，或者说他们不想和我呆在一起。 | 1 | 2 | 3 | 4 |
| 1. 我过得很好。 | 1 | 2 | 3 | 4 |
| 1. 我感到想哭。 | 1 | 2 | 3 | 4 |
| 1. 我感到悲哀。 | 1 | 2 | 3 | 4 |
| 1. 我觉得人们都不喜欢我。 | 1 | 2 | 3 | 4 |
| 1. 我觉得很难去开始做一件事情。 | 1 | 2 | 3 | 4 |

**MASC**

以下是一些对于**平时生活状态或内心状态的描述**。请你判断这些描述与你自己是否符合，并在在每个句子后面的1-5数字上圈选一个相应的数字。**“1”表示非常不符合你的情况，“5”表示非常符合。**

|  | 非常  不符合 | 比较不符合 | 不确定 | 比较符合 | 非常符合 |
| --- | --- | --- | --- | --- | --- |
| 1. 我担心别人会嘲笑我。 | 1 | 2 | 3 | 4 | 5 |
| 1. 我怕其他同学拿我取笑。 | 1 | 2 | 3 | 4 | 5 |
| 1. 我担心在课上被点到名字。 | 1 | 2 | 3 | 4 | 5 |
| 1. 我害怕其他人觉得我笨。 | 1 | 2 | 3 | 4 | 5 |
| 1. 我担心别人会怎么想我。 | 1 | 2 | 3 | 4 | 5 |
| 1. 我害怕我会做出一些愚蠢或让我感到尴尬的事情。 | 1 | 2 | 3 | 4 | 5 |
| 1. 喊其他同学一起玩对我来说是一件困难的事。 | 1 | 2 | 3 | 4 | 5 |
| 1. 当父母不在身边的时候我感到害怕。 | 1 | 2 | 3 | 4 | 5 |
| 1. 我很害怕和别人一起出去郊游。 | 1 | 2 | 3 | 4 | 5 |
| 1. 我晚上睡觉时会把灯开着。 | 1 | 2 | 3 | 4 | 5 |
| 1. 我不想在家人不在身边的情况下独自去什么地方。 | 1 | 2 | 3 | 4 | 5 |
| 1. 我尽量不看恐怖片。 | 1 | 2 | 3 | 4 | 5 |
| 1. 我坐在汽车（公交车或小汽车）里的时候会感到害怕。 | 1 | 2 | 3 | 4 | 5 |
| 1. 我对这些事物中的一种或几种感到害怕:坏天气、黑暗、高的地方、动物、虫子。 | 1 | 2 | 3 | 4 | 5 |
| 1. 我尽量和爸爸或妈妈呆在一起。 | 1 | 2 | 3 | 4 | 5 |
| 1. 我尽量远离那些让我心烦的事情。 | 1 | 2 | 3 | 4 | 5 |
| 1. 我尽我所能去顺从家长和老师。 | 1 | 2 | 3 | 4 | 5 |
| 1. 如果让我在公共场合表演，我会很紧张。 | 1 | 2 | 3 | 4 | 5 |
| 1. 我做事总是要先询问别人以得到许可。 | 1 | 2 | 3 | 4 | 5 |
| 1. 我总是先把所有事确认一遍。 | 1 | 2 | 3 | 4 | 5 |
| 1. 我睡觉时和家人（比如父母或外公外婆等）挨着一起睡。 | 1 | 2 | 3 | 4 | 5 |
| 1. 如果我感到烦心或害怕，我总是立刻显露出来。 | 1 | 2 | 3 | 4 | 5 |
| 1. 我总想要检查一下以确认所有事情都是安全的。 | 1 | 2 | 3 | 4 | 5 |
| 1. 我试图把所有事情都做到完全没有差错。 | 1 | 2 | 3 | 4 | 5 |

下面是一些对于**生理或心理感觉的描述**。请判断这些感觉是否经常发生在你身上，并且在每个句子后面的1-4数字上圈选一个相应的数字。**“1”表示从不发生，“4”表示经常发生**。

|  | 从不 | 偶尔 | 有时 | 经常 |
| --- | --- | --- | --- | --- |
| 1. 我感到紧张和焦躁 | 1 | 2 | 3 | 4 |
| 1. 我控制不住发抖 | 1 | 2 | 3 | 4 |
| 1. 我感到头昏或晕眩 | 1 | 2 | 3 | 4 |
| 1. 我感到神经紧张 | 1 | 2 | 3 | 4 |
| 1. 我感到心里隐隐作痛 | 1 | 2 | 3 | 4 |
| 1. 我感觉周围的世界不真实 | 1 | 2 | 3 | 4 |
| 1. 我的心跳过快或不稳 | 1 | 2 | 3 | 4 |
| 1. 我感到烦躁、坐立不安 | 1 | 2 | 3 | 4 |
| 1. 我的胃不舒服 | 1 | 2 | 3 | 4 |
| 1. 我的手发抖 | 1 | 2 | 3 | 4 |
| 1. 我的手出很多汗或发冷 | 1 | 2 | 3 | 4 |
| 1. 我感到呼吸困难 | 1 | 2 | 3 | 4 |
| 1. 我尽量睁着眼以便及时发现危险 | 1 | 2 | 3 | 4 |
| 1. 我感到害羞 | 1 | 2 | 3 | 4 |

**Suicide Ideation**

请阅读下面的一句话，**根据你自己的实际情况**，选择**“是”或“否”**。

| 在过去的两周之内，我曾有过自杀的念头，但没有付诸行动。 | 是 | 否 |
| --- | --- | --- |
